# Can Measuring the ‘Dual Anchors of Aorta’ Enhance the Success Rate of TAVR?—A Single-Center Experience

**DOI:** 10.3390/jcm12031157

**Published:** 2023-02-01

**Authors:** Yang Chen, Md Misbahul Ferdous, Lakshme Kottu, Jie Zhao, Hong-Liang Zhang, Mo-Yang Wang, Guan-Nan Niu, Qing-Rong Liu, Zheng Zhou, Zhen-Yan Zhao, Qian Zhang, De-Jing Feng, Bin Zhang, Zi-Ang Li, Daphne Merkus, Bin Lv, Hai-Yan Xu, Guang-Yuan Song, Yong-Jian Wu

**Affiliations:** 1 Department of Cardiology, Fuwai Hospital, National Center for Cardiovascular Disease, Chinese Academy of Medical Science and Peking Union Medical College, 167 Beilishilu, Xicheng District, Beijing 100037, China; 2 Department of Experimental Cardiology, Erasmus University Medical Center, 3015 CE Rotterdam, The Netherlands; 3 Walter-Brendel-Centre of Experimental Medicine, Ludwig-Maximilians-University München, 81377 Munich, Germany; 4 Department of Radiology, Fuwai Hospital, National Center for Cardiovascular Disease, Chinese Academy of Medical Science and Peking Union Medical College, 167 Beilishilu, Xicheng District, Beijing 100037, China; 5 Interventional Center of Valvular Heart Disease, Beijing Anzhen Hospital, Capital Medical University, Beijing 100029, China

**Keywords:** chronic aortic regurgitation, computed tomography anatomical classifications, transcatheter aortic valve replacement, transcatheter heart valve, leaflet calcification, root expansion

## Abstract

Introduction: Chronic severe aortic regurgitation (AR) has a poor long-term prognosis, especially among old-age patients. Considering their advancing age, the surgical approach of aortic valve replacement may not always be the best alternative modality of treatment in such patients. Therefore, this study’s primary goal was to provide an initial summary of the medium- and short-term clinical effectiveness of transcatheter aortic valve replacement (TAVR) guided by accurate multi-detector computed tomography (MDCT) measurements in patients with severe and chronic AR, especially in elderly patients. Methods: The study enrolled retrospectively and prospectively patients diagnosed with severe AR who eventually underwent TAVR procedure from January 2019 to September 2022 at Fuwai cardiovascular Hospital, Beijing. Baseline information, MDCT measurements, anatomical classification, perioperative, and 1-year follow-up outcomes were collected and analyzed. Based on a novel anatomical categorization and dual anchoring theory, patients were divided into four categories according to the level of anchoring area. Type 1, 2, and 3 patients (with at least two anchoring regions) will receive TAVR with a transcatheter heart valve (THV), but Type 4 patients (with zero or one anchoring location) will be deemed unsuitable for TAVR and will instead receive medical care (retrospectively enrolled patients who already underwent TAVR are an exception). Results: The mean age of the 37 patients with severe chronic AR was 73.1 ± 8.7 years, and 23 patients (62.2%) were male. The American Association of Thoracic Surgeons’ score was 8.6 ± 2.1%. The MDCT anatomical classification included 17 cases of type 1 (45.9%), 3 cases of type 2 (8.1%), 13 cases of type 3 (35.1%), and 4 cases of Type 4 (10.8%). The VitaFlow valve (MicroPort, Shanghai, China) was implanted in 19 patients (51.3%), while the Venus A valve (Venus MedTech, Hangzhou, China) was implanted in 18 patients (48.6%). Immediate TAVR procedural and device success rates were 86.5% and 67.6%, respectively, while eight cases (21.6%) required THV-in-THV implantation, and nine cases (24.3%) required permanent pacemaker implantation. Univariate regression analysis revealed that the major factors affecting TAVR device failure were sinotubular junction diameter, THV type, and MDCT anatomical classification (*p* < 0.05). Compared with the baseline, the left ventricular ejection fraction gradually increased, while the left ventricular end-diastolic diameter remained small, and the N-terminal-pro hormone B-type natriuretic peptide level significantly decreased within one year. Conclusion: According to the results of our study, TAVR with a self-expanding THV is safe and feasible for patients with chronic severe AR, particularly for those who meet the criteria for the appropriate MDCT anatomical classification with intact dual aortic anchors, and it has a significant clinical effect for at least a year.

## 1. Introduction

Among the elderly age group, aortic regurgitation (AR) is more common than aortic stenosis, as per a national survey conducted in China, and its prevalence is estimated to be around 1.2% [1,2,3,4]. Currently, surgical valve replacement is the standard treatment for chronic severe AR. However, some elderly patients are denied treatment due to their high surgical risk profile [5,6]. Transcatheter aortic valve replacement (TAVR) is a minimally invasive treatment to cure elderly patients with high-risk chronic severe AR.

## 2. Background

In recent years, TAVR has become increasingly popular as the preferred treatment of choice for patients with severe aortic stenosis [7]. While treating patients of severe AR with TAVR, there are still a number of challenges to be addressed. This is doubly important for patients who have surgical contraindications or who are at a very high risk [8,9,10]. The success rate of the latest generation of TAVR devices for treating AR is 89.9% [11,12], which is lower than that of the success rate of TAVR for treating aortic stenosis [13,14]. In China, the apical J-valve [15] and femoral artery self-expanding transcatheter heart valve (THV) [16,17] have been effective in treating AR. 

The difficulty of TAVR in treating AR stems from the absence of leaflet calcification and root expansion; therefore, the anchorage area of the AR anatomical structure is small, and therefore, there is high likelihood for THV displacement. However, detailed multi-detector computed tomography (MDCT) evaluation and screening of patients with chronic severe AR with appropriate anatomy can improve TAVR success rates [16,18]. Nonetheless, the current functional classifications of AR assessed using echocardiographs, which are associated with surgical valve replacement or repair and aortic root replacement [19,20], do not inform suitability for TAVR [21]. 

Therefore, our team proposed a novel AR dual-anchoring multi-planar measurement technique and MDCT anatomical classification [18,22]. Hence, the primary goal of this study was to outline the short- and medium-term outcomes of domestic TAVR with self-expanding THV in a single center, using MDCT high-precision measurements for treating patients with chronic severe AR. 

## 3. Materials and Methods

### 3.1. Study Population

**Figure 1:**
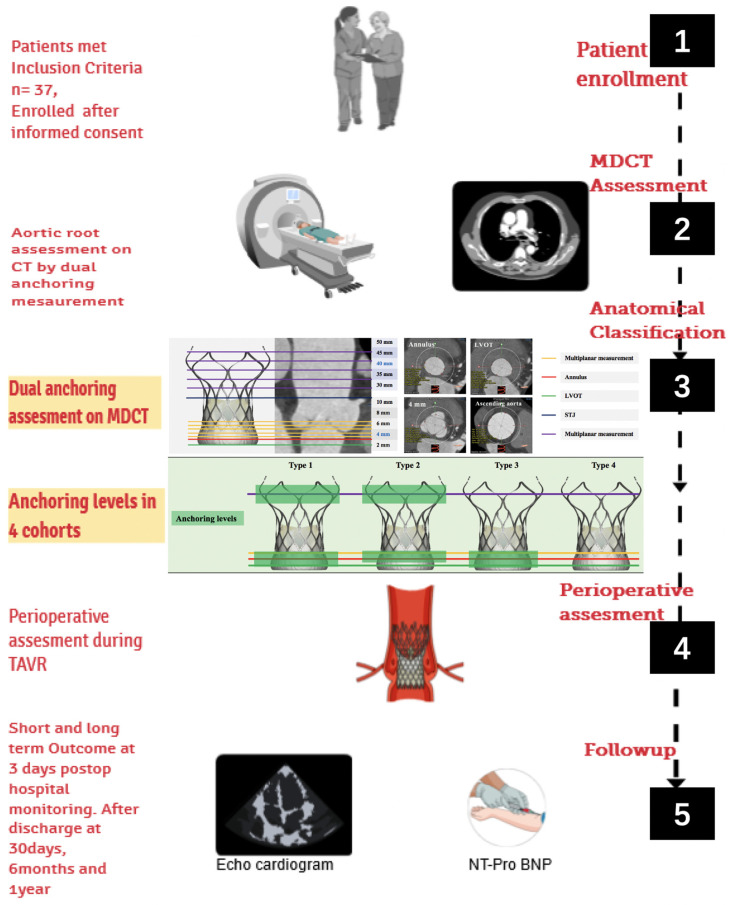
The study design overview of the medium- and short-term clinical effectiveness of TAVR guided by accurate MDCT measurements in patients with chronic severe AR. TAVR = transcatheter aortic valve replacement; MDCT = multi-detector computed tomography; AR = aortic regurgitation.

Between January 2019 and July 2020, data were acquired retrospectively, and between July 2020 and September 2022, data were collected prospectively. Inclusion criteria and exclusion criteria are shown in Table 1. All patients provided written informed consent for TAVR and the collection of follow-up data, as shown in the study design overview (Figure 1). The following criteria were used to define severe native AR in the 2017 American Society of Echocardiography guidelines: (1) vena contracta width > 6 mm; (2) pressure half-time < 200 ms; (3) effective regurgitant orifice area ≥ 30 mm^2^; (4) regurgitant volume ≥ 60 mL/beat; and (5) left ventricle dilation [18,23,24].

### 3.2. MDCT Evaluation and Anatomical Classification

All patients who were enrolled with chronic severe AR in this study underwent a full-time MDCT angiography of the aortic root with a 64-slice dual-source multi-detector computed tomography (MDCT) (Somatom Definition or Somatom Force, Siemens Healthcare, Forchheim, Germany). A special software program was used for the analysis of these TAVR MDCT datasets (3mensio, Pie Medical, Maastricht, The Netherlands) [25]. MDCT measurement parameters included the number of aortic valve leaflets, annulus, left ventricular outflow tract (LVOT), sinotubular junction (STJ), ascending aorta (AA), and calcification score [26]. Dual-anchoring multiplane measurement included the diameter calculated at 2 mm below the annulus, and 2, 4, 6, 8, 10, 30, 35, 40, 45, and 50 mm above the annulus [18,22]. According to the dual-anchoring theory, the annulus and supra-annular structures are the first major anchoring areas, the LVOT and 2 mm below the annulus are the second major anchoring areas, and the STJ and AA (30, 35, 40, 45, and 50 mm above the annulus and the widest part) are secondary anchoring areas [18,22]. Patients were deemed to have the proper anatomy for TAVR treatment if they had either the first major anchoring area or the second major or at least secondary anchoring sites during their MDCT evaluation. These patients with chronic severe AR were then separated into four types, of which types 1–3 were considered to be appropriate for treatment with TAVR, and type 4 maybe not ideal for TAVR (Figure S1). In type 1 patients, THV was the most stable after release and deployment because type 1 patients can anchor the annulus, LVOT, and AA area and also its observed that the anchoring area is longest. Due to the large diameter of the LVOT of type 2, the THV frame is less stable before and after THV deployment than that of type 1. Type 3 is AA dilatation; this type can only be anchored in the LVOT and annulus area. After implantation, the THV frame’s stability was less stable than that of types 1 and 2. It is not recommended to use TAVR for type 4 because the annulus, LVOT, and AA area are too large to be anchored [18,22]. Four patients with type 4 were retrospectively collected according to the novel anatomical classification, while the patients with type 4 were not recommended for TAVR in the prospective enrollment. In this study, the anchorage area of patients with AR was defined to meet the oversize rate of >10%.

Oversize ratio = 100 × (THV diameter/MDCT measurement diameter − 1)

### 3.3. Operation

The hybridized catheter room is where the TAVR operations were performed either under local or general anesthesia, decided on a case-by-case basis. A multidisciplinary cardiac team was involved in the procedure, and the team members included interventional cardiologists, cardiac surgeons, cardiologists from imaging/echocardiogram units, nurses, and anesthesiologists. The two China-made self-expanding THVs used in this study were Venus A Valve (Venus MedTech, Hangzhou, China) [27] and VitaFlow Valve (MicroPort, Shanghai, China) (Figure S2) [28]. It was not necessary to pre-expand the balloon; however, it was essential to pace the heart at 180 times/min to ensure that short-term blood ejection ability was ceased and that the THV release process was stable. The THV release was conducted in two steps: the first release after ensuring that the anchor annulus of the THV and the LVOT had been stabilized, and the second release was carried out when pacing ceased. The THV-in-THV strategy was applied if there was a displacement of the first THV after implantation, leading to significant perivalvular leakage.

### 3.4. Perioperative Outcome and Follow-Up

The ‘immediate device success rate’ was defined as a single THV completing TAVR successfully (excluding THV-in-THV implantation), and the THV position was appropriate. There was no significant perivalvular leakage determined by echocardiography due to good THV function, along with normal flow rate, and mild or no pressure difference. The ‘immediate surgical success rate’ was defined as THV implantation (including THV-in-THV implantation) without serious complications, such as annular rupture, coronary occlusion, aortic root dissection, and access injury (dissection, rupture, and bleeding). According to the Valve Academic Research Consortium-3 consensus criteria, all-cause death, cardiovascular death, life-threatening hemorrhage, stroke, acute renal injury (phase 3), coronary artery occlusion requiring interventional treatment, major vascular complications, perivalvular leakage, and permanent pacemaker implantation within one month were recorded [29]. The working status of the THV (echocardiography analysis of valve thrombus, perivalvular leakage, and THV displacement) and the incidence of all-cause death, major cardiovascular events, stroke, and heart failure readmission in one year were collected.

### 3.5. Statistical Analysis

The measurement data were expressed as means ± standard deviations and compared using the unpaired Student’s *t*-test or Mann–Whitney U test and analysis of variance. The paired Student’s *t*-test or Wilcoxon signed-rank test was used comparing consecutive values between baseline, postoperative, and follow-up data. Categorical variables were presented as numbers (proportions), and the practical χ2 test or Fisher’s exact probability test was used to determine the significance of different groups. Variables with *p* < 0.05 in the univariate logistic analysis were included in the multivariate logistic regression model for analysis. Statistical significance was set at *p* < 0.05. All data were statistically analyzed using SPSS 24.0 software. Graphs were created using ggplot2, plotly packages of R4.0.5 (The R project for statistical computing, Vienna, Austria).

## 4. Results

### 4.1. Basic Characteristics of Patients with Chronic Severe AR Undergoing TAVR Treatment

**Figure 2:**
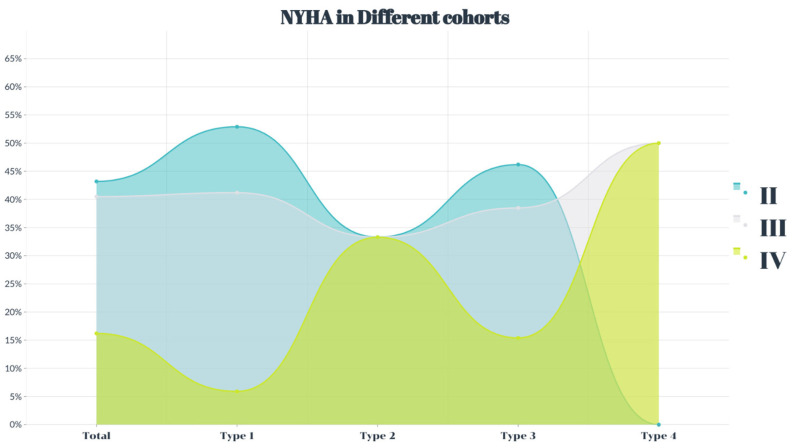
The percentage of the New York Heart Association function grade in severe aortic regurgitation.

This study included 37 patients with an average age of 73.1 ± 8.7 years. The study included 23 male patients (62.2%). All patients had heart failure symptoms (Figure 2), and the average Society of Thoracic Surgeons score was 8.6 ± 2.1%. The age of type 4 patients was older than that of the participants in the other three cohorts (*p* < 0.05), and the four groups showed no differences in other clinical complications (*p* > 0.05) (Table 2).

### 4.2. Preoperative MDCT Anatomical Characteristics

Thirty-three patients (89.2%) had tricuspid valves, with only three (8.1%) having mild leaflet calcification. No significant difference in the circumference or diameter of the annulus was observed among the four groups (*p* < 0.05). The LVOT and 2 mm diameter below the annulus in type 4 patients were larger than those in types 1 and 3 (*p* < 0.05). However, there was no difference among the 4, 6, 8, and 10 mm diameters above the annulus. There were differences in STJ, AA (30–50 mm and the widest), and diameter of the three sinuses among the four groups (*p* < 0.05). The difference was mainly due to the obvious ascending dilatation in type 3 patients (Table 3).

### 4.3. Operation Outcome and Device Failure Analysis

**Figure 3:**
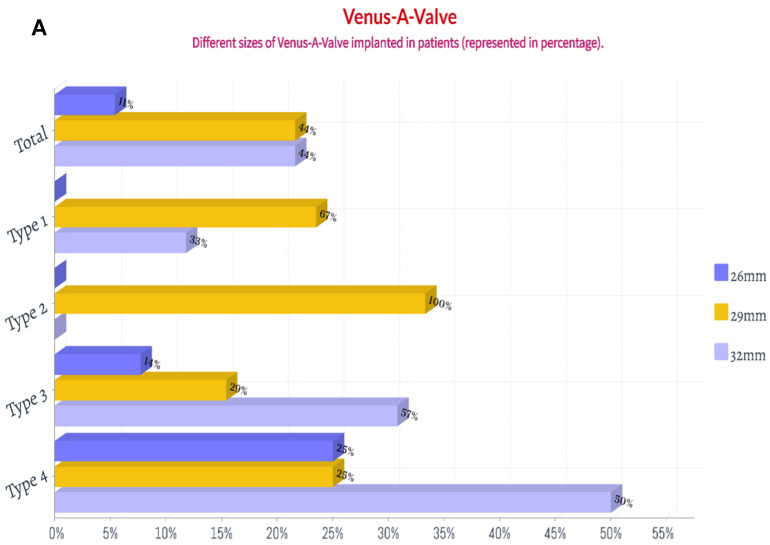

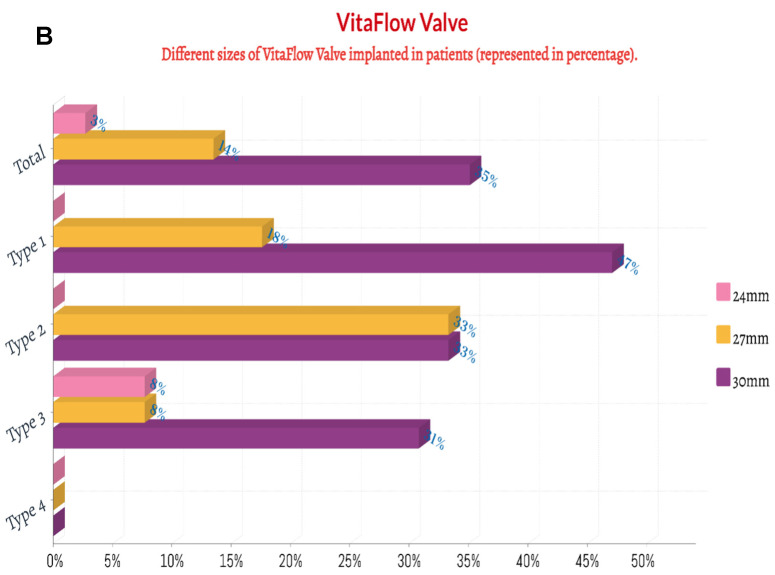
The percentage of the implanted size of Venus A valve and VitaFlow valve in patients with chronic severe aortic regurgitation. (**A**): Venus A valve; (**B**): VitaFlow valve.

**Figure 4:**
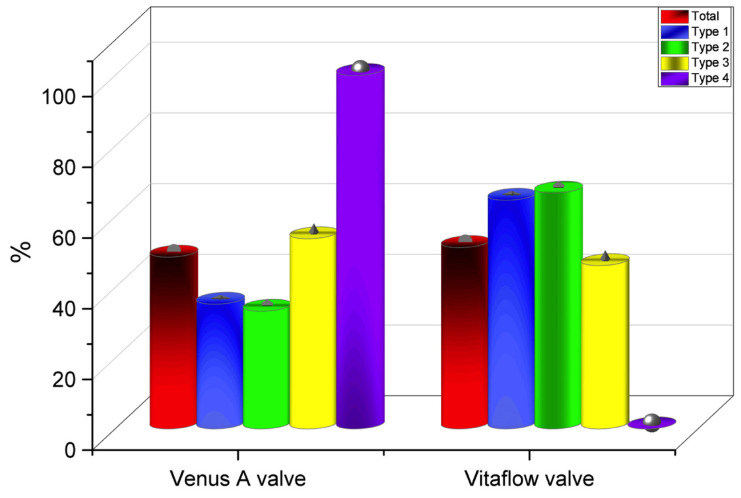
Total number of valves implanted (in percentages) in all groups.

Thirty-six patients (97.3%) underwent the femoral artery approach, while only one (2.7%) underwent the carotid artery approach. Twenty-one patients (56.8%) before May 2021 received general anesthesia, while sixteen (43.2%) after May 2021 received local anesthesia alone. Twenty-eight patients (75.7%) before Aug 2021 had first-generation non-recyclable THV systems, while the remaining nine (24.3%) after Aug 2021 had second-generation recyclable THV systems. Eighteen patients (48.6%) were implanted with Venus A valve, while nineteen patients (51.3%) were implanted with the VitaFlow valve (Figure 3 and Figure 4). There was no difference in these characteristics among the four groups (*p* > 0.05) (Table 4).

In type 1 patients, the annulus, narrowest position above the annulus, LVOT, 2 mm below the annulus, AA, and narrowest position of AA were more than 10% of the narrowest positions, with 20.4 ± 8.7%, 16.7 ± 6.7%, 17.9 ± 10.8%, 20.3 ± 9.6%, 17.5 ± 9.9%, and 25.8 ± 20.7%, respectively. Compared with type 1 patients, type 2 patients had a significantly lower annulus, LVOT, and 2 mm below the annulus (*p* < 0.05). Compared with type 1 patients, type 3 patients had significantly lower AA and the narrowest AA overlap (*p* < 0.05). Compared with type 1 patients, type 4 patients had a significantly lower annulus, LVOT, 2 mm below the annulus, AA, and the narrowest AA area (*p* < 0.05).

**Figure 5:**
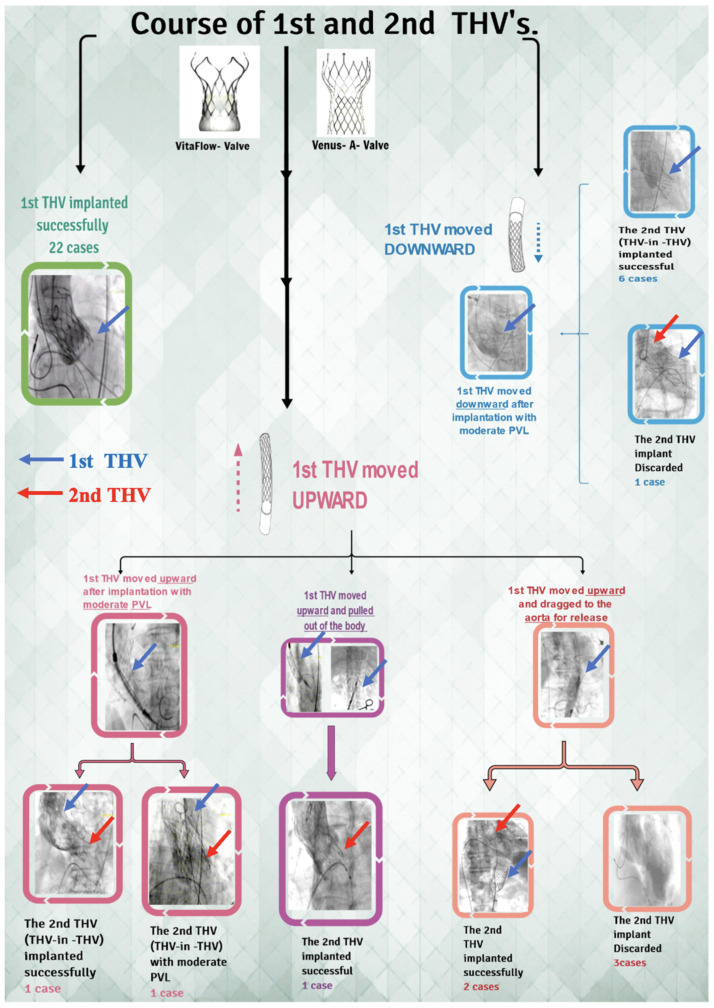
The flowchart of TAVR procedure and immediate outcome in chronic severe AR. THV = transcatheter heart valve; PVL = perivalvular leakage; TAVR = transcatheter aortic valve replacement; AR = aortic regurgitation.

**Figure 6:**
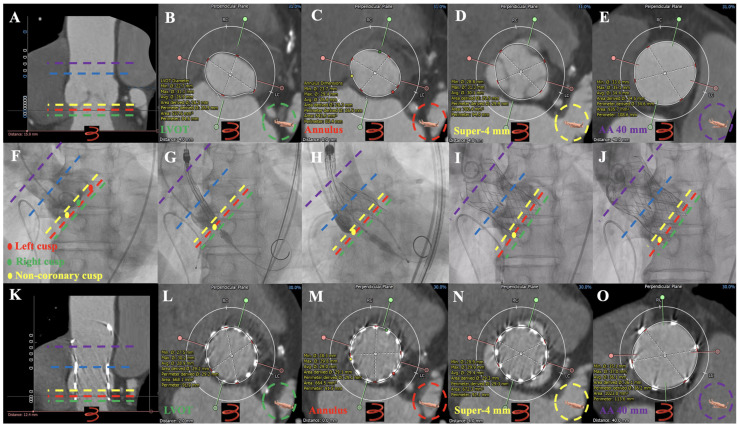
Dual anchoring measurements in Preoperative MDCT, Operative Fluro, Follow-up MDCT images in one case of severe AR underwent TAVR guided using MDCT anatomical classifications: type 1. Preoperative MDCT: (**A**–**E**): aortic root imaging at longitudinal section, LVOT, annulus, super-annular 4 mm, and AA 40 mm in patients with type 1. Operative Fluro: (**F**)*:* aortic root angiography; (**G**)*:* initial positioning of 30 mm VitaFlow valve; (**H**): half-releasing at 180 bpm (pacing lead in right ventricular); (**I**,**J**): VitaFlow valve deployed completely. Follow-up MDCT: (**K**–**O**): aortic root imaging at longitudinal section, LVOT, annulus, super-annular 4 mm, and AA 40 mm in patients with 30 mm VitaFlow valve implantation. ● = Left Coronary cusp; ● = Right Coronary cusp; ● = Non-coronary cusp. ▬ ▬ ▬ ▬ = Annulus; ▬ ▬ ▬ ▬ = LVOT; ▬ ▬ ▬ ▬ = super-annular 4 mm; ▬ ▬ ▬ ▬  = AA 40 mm; ▬ ▬ ▬ ▬ = sinotubular junction. AR = aortic regurgitation; TAVR = transcatheter aortic valve replacement; MDCT = multidetector computed tomography; AA = ascending aorta.

**Figure 7:**
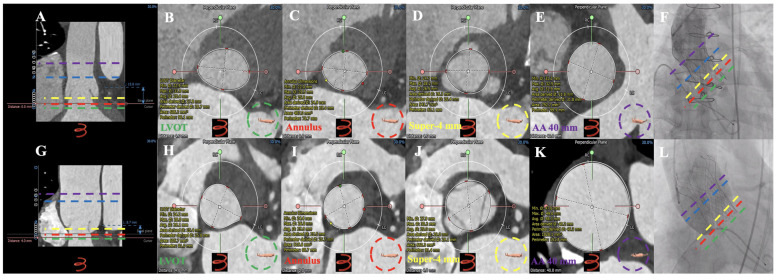
MDCT measurements and successful TAVR in type 2 and type 3 patients with chronic severe AR. CT anatomical classifications—Type 2: (**A**): longitudinal section of the aortic root; (**B**): LVOT; (**C**): annulus; (**D**): super-annular 4 mm; (**E**): AA 40 mm; (**F**): 27 mm Vitaflow valve successfully implanted. CT anatomical classifications—Type 3: (**G**): longitudinal section of the aortic root; (**H**): LVOT; (**I**): annulus; (**J**): 4 mm above the annulus; (**K**): AA 40 mm; (**L**): 30 mm Vitaflow valve successfully implanted. ▬ ▬ ▬ ▬ = Annulus; ▬ ▬ ▬ ▬ = LVOT; ▬ ▬ ▬ ▬ = super-annular 4 mm; ▬ ▬ ▬ ▬  = AA 40 mm; ▬ ▬ ▬ ▬ = sinotubular junction. AR = aortic regurgitation; TAVR = transcatheter aortic valve replacement; MDCT = multidetector computed tomography; LVOT = left ventricular outflow tract; AA = ascending aorta.

**Figure 8:**
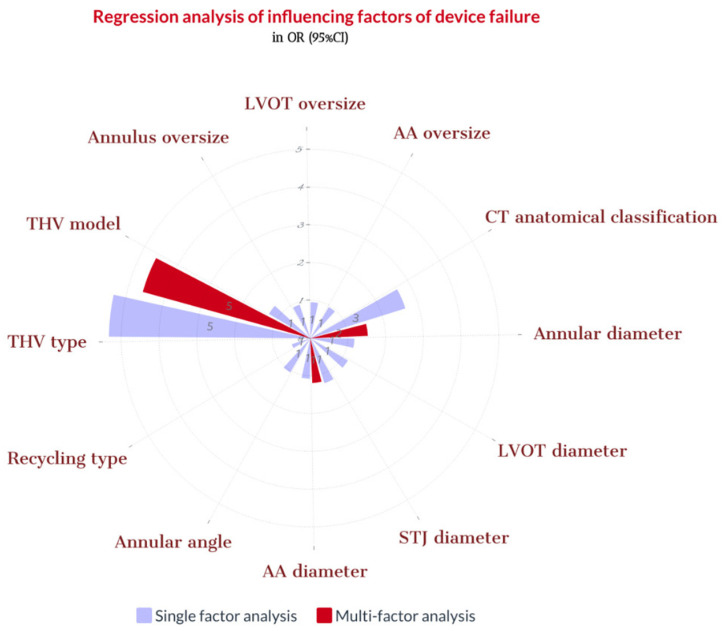
Regression analysis of influencing factors of device failure. Note: CT = computed tomography; LVOT = left ventricular outflow tract; THV = transcatheter heart valve; STJ = sinotubular junction; AA = ascending aorta. Oversize = 100 × (THV diameter/MDCT measurement diameter − 1).

During the procedure, we noticed that in six patients (16.2%), THV slipped down immediately after release, and in eight patients (21.6%), THV moved upward after the release. One patient’s THV was pulled out of the body, and five patient’s THV was released into the aorta. Finally, the second THV (THV-in-THV) was successfully implanted in eight patients without any perivalvular leakage, while two patient’s echoes showed moderate perivalvular leakage. Subsequently, three patients declined the second THV implantation (Figure 5). Ultimately, 25 cases (67.6%) of immediate devices and 32 cases (86.5%) of operations were successful (Figure 6 and Figure 7). The implantation rate of THV-in-THV and the success rate of the devices differed significantly between the four groups (*p* < 0.05); however, the surgical success rate did not differ (*p* > 0.05). The single-factor regression analysis revealed that the main influencing factors of the display failure rate were STJ diameter, THV type, and MDCT anatomical classification (*p* < 0.05), but the multiple-factor regression analysis revealed no statistical difference (*p* > 0.05) (Figure 8).

Nine patients (24.3%) during their post-op recovery days in the hospital underwent permanent pacemaker implantation as they developed a third-degree atrioventricular block. Two cases (5.4%) of thoracic aortic dissection were treated with covered stent implantation through vascular surgery. No annular rupture, serious life-threatening or severe bleeding, or valve embolism was observed. There were no other perioperative complications, such as myocardial infarction, pericardial effusion, vascular access complications, bleeding, hypotension, ventricular wall rupture, or acute renal failure.

### 4.4. Follow-Up Outcomes

During the one-year follow-up period, two deaths were reported, one of whom died one month after TAVR failure and the other 11 months after successful THV-in-THV implantation due to cardiac arrest.

Nine months after discharge, one patient had TAVR failure evident with moderate perivalvular leakage, necessitating repeated heart failure hospitalizations. In addition, one patient with TAVR failure received a J-valve implant using the apical approach. The other two patients with TAVR failure were treated conservatively, and their conditions stabilized.

A type 3 patient had a THV implanted successfully, but after three months, it moved down, causing significant perivalvular leakage; thus, a second THV (THV-in-THV) was implanted. No moderate to severe perivalvular leakage was observed in other patients in this cohort. Two patients from the type 1 cohort had transient ischemic attacks within one month after the operation and improved after drug treatment. No additional adverse events, e.g., cardiac conduction abnormalities such as ventricular tachyarrhythmias or new onset LBBB, major (disabling) stroke, or life-threatening bleeding, were observed.

**Figure 9:**
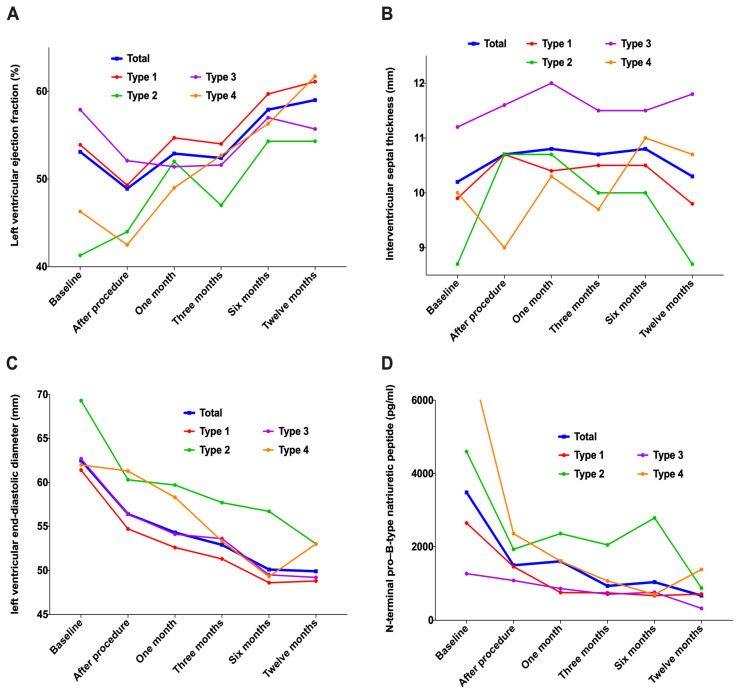
Changes in cardiac morphology and function during 1-year follow-up. (**A**): Left ventricular ejection fraction; (**B**): Interventricular septal thickness; (**C**): Left ventricular end-diastolic diameter; (**D**): N-terminal pro–B-type natriuretic peptide.

In addition to three patients with TAVR failure who did not have THV implanted at the THV position, the analysis of the remaining 34 patients revealed that the left ventricular ejection fraction (LVEF) gradually increased compared with the baseline within one year after TAVR; however, the change in ventricular septal thickness was stable, the diameter of the left ventricle at the end of diastole showed significant decline along with N-terminal pro–B-type natriuretic peptide (NT-ProBNP) (Figure 9 and Table 5).

## 5. Discussion

Guidelines recommend that mature TAVR centers select patients with suitable anatomy for treatment [9,10]. In this study, patients with chronic severe AR of types 1–3 with appropriate anatomy were selected after an accurate evaluation of the MDCT dual-anchoring multi-planar measurement scheme, and TAVR was performed using a ‘made in China’ self-expanding THV. Although surgical success rates were high, the proportion of THV-in-THV and pacemaker implantation rates remained high. Following a year of monitoring, patients’ cardiac function gradually improved. 

Most of the patients in this study who underwent TAVR were elderly patients with chronic severe AR who were at high surgical risk. MDCT revealed that the aortic valve was mainly tricuspid, while the quadricuspid valve and type 1 bicuspid valve accounted for a small portion. The supravalvular structure of patients with quadricuspid valves was almost anchored, while the supravalvular structure of patients with type 1 bicuspid valves may have an anchored area due to the congenital adhesion of two leaflets. The multi-planar measurement method used to pre-evaluate the THV can help to better understand the supravalvular structure, such as the anchoring area of leaflet adhesion, in order to determine the length of the anchored area and to further aid its release more steadily. Therefore, aortic root anatomical classification for patients with chronic severe AR based on the size of the implanted THV and MDCT dual-anchoring multi-planar measurements is imperative. However, this is preoperative virtual computing, though, as the depth of THV implantation can greatly impact anatomical classification and accuracy. A first-generation THV delivery system was used mainly in the initial phase of this study, which had a low non-recyclable fault tolerance rate. However, the second-generation recyclable system was able to enhance implantation stability and success rates by maximizing the implantation depth.

In this study, no difference was observed between the success rate of the THV device used and routine MDCT measurements, such as the annulus, LVOT, AA, and its override. This was due to the patients who were included in the study having undergone a preliminary screening for suitable anatomy. The main factors influencing the device failure rate are the MDCT anatomical classification, STJ diameter, and THV types. That revealed that the area anchored by the annulus and LVOT is relatively short; hence, the implanted THV release is not sufficiently stable. Therefore, evaluating the anchoring effect of the STJ and AA in patients with chronic severe AR is necessary. The THV types are mainly due to the different designs of the two THVs. The straight cylindrical frame of the VitaFlow valve causes the THV frame to move with the same tension, making the THV relatively stable after release. In addition, the VitaFlow has an outer skirt design, which increases the friction between the THV and surrounding tissues, effectively preventing the THV frame from moving downward (Figure S2) [28]. The Venus A valve waist retraction design has advantages in treating patients with aortic stenosis because it reduces the tension caused by severe leaflet calcification and leaflet movement to the sinus wall, lowering the risk of coronary artery occlusion (Figure S2) [27]. However, the waist retraction design of the Venus A valve is unsuitable for patients with chronic severe AR. The waist retraction design causes instability of the THV position because the leaflet of patients with AR has no calcification or support force, leading to downward movement.

In patients with an inappropriate release position, the THV may be displaced, which is why implanting a second THV is essential to reduce perivalvular leakage. Moreover, the incidence of THV implantation is high. However, late displacement of the implanted THV and perivalvular leakage should be monitored continuously, and TAVR or perivalvular leakage closure may be required to reduce moderate and large perivalvular leakages. In this study, two cases of late thoracic aortic dissection occurred, which could be related to the excessive tension of the THV delivery system and the widening and thinning of the aortic wall in patients with chronic severe AR. The implantation rate of permanent pacemakers is very high because of the excessive stress on the annulus and LVOT anchorage area in patients with chronic severe AR. In addition, according to the current domestic THV types and models, patients with type 4 cannot undergo TAVR. There are many design concepts specifically for AR treatment with THV [30]. Therefore, designing prosthetic THV systems for patients with chronic severe AR is necessary to solve the problem of minimally invasive TAVR treatment.

LVEF increased progressively during a 1-year follow-up period, indicating that reverse cardiac remodeling in patients with chronic severe AR was slow, which was different from that of LVEF recovery, and reversed cardiac remodeling in patients with aortic stenosis after TAVR. In addition, LVEF decreased immediately after TAVR because of the increase in LVEF in patients with chronic severe AR during the compensatory period before surgery [31]. In contrast, the compensatory volume LVEF increased and disappeared due to the immediate contraction of the ventricle in patients with chronic severe AR after surgery. This study revealed that the interventricular septal thickness changed a little, but the left ventricular end-diastolic shortened significantly and continuously, indicating that the pathophysiological process of left ventricular volume passive expansion after TAVR was completely cut off [32,33]. In addition, NT-ProBNP levels decreased significantly to normal levels gradually, indicating that heart failure in patients after TAVR improved gradually.

This study had some limitations. Firstly, as this is a single-center study, it had a small sample size. Secondly, as we are submitting early findings, the follow-up period is shorter, thus we look forward and anticipate significant long-term outcomes from the ongoing multicenter trial (AURORA study, ChiCTR2200055415) [22]. Third, the anatomical classification was significantly related to MDCT measurement and THV type as well as size, which is affected by the true implantation depth of THV. This study’s analysis of MDCT-guided THV implantation depth after TAVR, has high requirements for the operator’s experience in TAVR operation, therefore challenging to promote the technique in a wider horizon.

## 6. Conclusions

For TAVR treatment, selecting patients with appropriate MDCT anatomical AR classifications and self-expanding, THV is safe and effective, with significant short-term effects and gradual recovery of cardiac morphology and function. In order to verify the long-term effectiveness of TAVR as the treatment of choice in patients with chronic severe AR and the effects of specific THV devices for AR, large-sample clinical trials are currently required. The success of TAVR in patients with intact anchors of the aorta is encouraging, but there is still a long way to explore how to make it successful in patients with poor ‘dual anchors’ of the aorta.

## Figures and Tables

**Table 1:** Inclusion criteria and exclusion criteria.

Standards	Contents
Inclusion criteria	
	Age ≥ 55 years
	Symptomatic severe AR
	American Association of Chest Physicians score > 4%
	New York Heart Association grades II–IV
	MDCT evaluation anatomy appropriate for TAVR treatment
Exclusion criteria	
	Acute myocardial infarction within 30 days
	Left ventricular ejection fraction ≤ 20%
	Life expectancy < 1 year
	Mild to moderate AR
	With moderate to severe aortic stenosis
	Previous aortic valve replacement
	Inappropriate MDCT evaluation anatomy
	Other THV treatments

Note: AR = aortic regurgitation; MDCT = multi-detector computed tomography; TAVR = transcatheter aortic valve replacement; THV = transcatheter heart valve.

**Table 2:** Basic characteristics of patients with severe aortic regurgitation.

Characteristics (n, %)	Total N = 37	Type 1 N = 17	Type 2 N = 3	Type 3 N = 13	Type 4 N = 4	*p*
Age	73.1 ± 8.7	71.4 ± 7.2	67.7 ± 11.0	73.7 ± 9.9	82.3 ± 4.3 ^abc^	0.096
Male	23 (62.2)	10 (58.8)	3 (100.0)	7 (53.8)	3 (75.0)	0.463
STS score (%)	8.6 ± 2.1	7.9 ± 1.9	7.7 ± 0.8	9.2 ± 2.2	10.2 ± 2.5	0.123
Coronary artery disease	14 (37.8)	6 (35.3)	3 (100.0)	4 (30.8)	1 (25.0)	0.137
Previous PCI	3 (8.1)	2 (11.8)	1 (33.3)	0	0	0.224
Previous CABG	2 (5.4)	1 (5.9)	1 (33.3)	0	0	0.135
Atrial fibrillation	13 (35.1)	6 (35.5)	1 (33.3)	4 (30.8)	2 (50.0)	0.919
Previous PPI	2 (5.4)	1 (5.9)	0	1 (7.7)	0	0.910
hypertension	23 (62.2)	9 (52.9)	2 (66.7)	11 (84.6)	1 (25.0)	0.123
Hyperlipidemia	21 (56.8)	9 (52.9)	2 (66.7)	8 (61.5)	2 (50.0)	0.937
Diabetes mellitus	7 (18.9)	7 (11.8)	2 (66.7)	2 (15.4)	1 (25.0)	0.156
COPD	3 (8.1)	2 (11.8)	0	0	1 (25.0)	0.355
Cerebral vascular disease	2 (5.4)	1 (5.9)	1 (33.3)	0	0	0.135
Hepatic insufficiency	2 (5.4)	1 (5.9)	1 (33.3)	0	0	0.135
Peripheral vascular disease	2 (5.4)	1 (5.9)	0	1 (7.7)	0	0.910

Note: STS = Society of Thoracic Surgeons; PCI = percutaneous coronary intervention; CABG = coronary artery bypass grafting; PPI = permanent pacemaker implantation; COPD = chronic obstructive pulmonary disease. a: comparison between types 2–4 and type 1; b: comparison between types 3–4 and type 2; c: comparison between types 4 and 3, *p* < 0.05.

**Table 3:** MDCT measurement.

Characteristics (n, %)	Total N = 37	Type 1 N = 17	Type 2 N = 3	Type 3 N = 13	Type 4 N = 4	*p*
Valvular classification						
Tricuspid valve	33 (89.2)	16 (94.1)	3 (100.0)	11 (84.6)	3 (75.0)	0.574
Quadricuspid valve	2 (5.4)	1 (5.9)	0	1 (7.7)	0	
Bicuspid valve	2 (5.4)	0	0	1 (7.7)	1 (25.1)	
Measurement (diameter calculated by the perimeter, mm)						
Annular circumference	81.7 ± 6.8	80.6 ± 5.2	85.3 ± 4.7	80.8 ± 7.1	86.9 ± 11.3	0.280
Annulus	26.0 ± 2.2	25.6 ± 1.7	17.1 ± 1.5	15.7 ± 2.3	27.7 ± 3.6	0.284
LVOT	26.9 ± 2.9	26.2 ± 2.0	28.8 ± 2.4	26.5 ± 2.8	30.1 ± 4.9 ^ac^	0.053
STJ Height	25.67 ± 5.4	24.0 ± 3.2	30.9 ± 4.7 ^a^	27.5 ± 7.1	23.2 ± 4.0	0.078
STJ	35.7 ± 4.9	32.4 ± 2.7	35.1 ± 4.9	39.9 ± 4.7 ^a^	36.3 ± 2.8	0.000
AA widest	41.9 ± 6.2	38.6 ± 4.6	38.0 ± 4.7	47.2 ± 5.3 ^ab^	41.8 ± 5.5	0.000
Right coronary sinus	36.9 ± 4.5	35.2 ± 3.2	39.5 ± 7.9	38.6 ± 4.5 ^a^	36.6 ± 5.8	0.156
Left coronary sinus	37.6 ± 4.3	35.5 ± 2.9	42.1 ± 4.3 ^a^	39.4 ± 4.3 ^a^	37.5 ± 6.0	0.019
Non-coronary sinus	38.2 ± 4.1	36.3 ± 2.8	41.4 ± 5.8 ^a^	40.1 ± 4.4 ^a^	37.8 ± 3.7	0.037
Left coronary height	14.4 ± 4.0	14.3 ± 3.6	20.4 ± 4.2 ^a^	13.7 ± 3.7	12.7 ± 4.7	0.045
Right coronary height	18.6 ± 3.4	18.1 ± 3.0	22.3 ± 3.5	18.4 ± 4.0	19.0 ± 2.6	0.268
Annulus angle	49.7 ± 12.4	48.2 ± 9.0	49.3 ± 3.5	51.6 ± 17.4	50.3 ± 12.3	0.910
>60°	7 (18.9)	1 (5.9)	0	5 (38.5)	1 (25.0)	0.092
50°–60°	12 (32.4)	4 (23.5)	1 (33.3)	6 (46.2)	1 (25.0)	
<50°	18 (48.6)	12 (70.6)	2 (66.7)	2 (15.4)	2 (50.0)	
Dual-anchoring multiplane measurement (diameter calculated by the perimeter, mm)						
Sup-2 mm	26.2 ± 2.6	25.7 ± 1.7	27.5 ± 1.7	25.5 ± 2.3	29.3 ± 5.0 ^ac^	0.040
Sup-2 mm	26.7 ± 2.0	26.4 ± 1.6	27.9 ± 1.4	26.7 ± 2.3	26.8 ± 2.9	0.715
Sup-4 mm	28.3 ± 2.1	28.2 ± 1.8	29.4 ± 2.0	28.4 ± 2.5	27.0 ± 2.4	0.516
Sup-6 mm	29.6 ± 2.2	29.4 ± 2.0	31.1 ± 2.0	29.8 ± 2.2	28.0 ± 2.8	0.295
Sup-8 mm	30.5 ± 2.4	30.1 ± 1.7	31.8 ± 4.5	31.1 ± 2.5	28.8 ± 2.9	0.254
Sup-10 mm	31.2 ± 2.6	30.6 ± 1.8	32.4 ± 5.0	32.2 ± 2.6	30.0 ± 3.0	0.212
Sup-min	26.6 ± 2.0	26.4 ± 1.7	27.9 ± 1.4	26.7 ± 2.3	26.2 ± 2.9	0.686
AA-30 mm	37.7 ± 5.4	34.2 ± 2.8	36.1 ± 5.8	42.5 ± 5.2 ^ab^	38.5 ± 2.6	0.000
AA-35 mm	38.7 ± 5.4	35.3 ± 2.9	35.3 ± 4.0	43.6 ± 5.4 ^ab^	39.0 ± 2.7 ^c^	0.000
AA-40 mm	39.7 ± 5.4	36.6 ± 3.1	36.2 ± 3.8	44.6 ± 5.3 ^ab^	39.5 ± 3.4	0.000
AA-45 mm	40.6 ± 5.6	37.5 ± 3.6	36.9 ± 3.9	45.5 ± 5.4 ^ab^	40.7 ± 3.2	0.000
AA-50 mm	41.6 ± 6.1	38.0 ± 4.1	37.7 ± 3.8	46.3 ± 5.4 ^ab^	44.6 ± 5.9 ^a^	0.000
AA-min	37.5 ± 5.2	34.2 ± 2.8	34.9 ± 4.7	42.2 ± 4.8 ^ab^	37.9 ± 2.3	0.000

Note: MDCT = multidetector computed tomography; LVOT = left ventricular outflow tract; STJ = sinotubular junction; AA = ascending aorta. a: comparison between types 2–4 and type 1; b: comparison between types 3–4 and type 2; c: comparison between types 4 and 3, *p* < 0.05.

**Table 4:** Operation process and treatment outcome.

Characteristics (n, %)	Total N = 37	Type 1 N = 17	Type 2 N = 3	Type 3 N = 13	Type 4 N = 4	*p*
Retrospective cases	7 (18.9)	0	0	3 (23.1)	4 (100.0)	0.000
Prospective cases	30 (81.1)	17 (100.0)	3 (100.0)	10 (76.9)	0	
General anesthesia	21 (56.8)	10 (58.8)	3 (100.0)	7 (53.8)	1 (25.0)	0.261
Local anesthesia *	16 (43.2)	7 (41.2)	0	6 (46.2)	3 (75.0)	
First-generation THV	28 (75.7)	12 (70.6)	3 (100.0)	9 (69.2)	4 (100.0)	0.426
Second-generation THV ^#^	9 (24.3)	5 (29.4)	0	4 (30.8)	0	
Oversize (%)						
Annulus	17.4 ± 7.9	20.4 ± 8.7	10.6 ± 1.7 ^a^	18.0 ± 4.8	8.0 ± 5.2 ^a^	0.009
Narrowest super-annulus	14.7 ± 7.4	16.7 ± 6.7	7.7 ± 4.5	13.7 ± 7.1	13.9 ± 11.0	0.240
LVOT	13.9 ± 11.3	17.9 ± 10.8	4.3 ± 5.0 ^a^	15.2 ± 9.6	−0.4 ± 7.5 ^ac^	0.007
2 mm below the annulus	17.1 ± 9.9	20.3 ± 9.6	9.3 ± 3.3 ^a^	19.0 ± 6.8	2.6 ± 8.2 ^a^	0.002
Ascending aorta	8.3 ± 15.2	17.5 ± 9.9	19.8 ± 13.3	−5.1 ± 11.2 ^ab^	4.0 ± 14.3 ^ab^	0.000
Narrowest AA	14.7 ± 16.6	25.8 ± 20.7	25.0 ± 18.4	−0.0 ± 11.5 ^ab^	7.8 ± 10.9 ^a^	0.000
Outcome						
THV downward	6 (16.2)	1 (5.9)	2 (66.7)	2 (15.4)	1 (25.0)	0.066
THV upward	8 (21.6)	1 (5.9)	0	5 (38.5)	2 (50.0)	0.060
THV release into the artery	5 (13.5)	1 (2.7)	0	3 (23.1)	1 (25.0)	0.426
THV pulled out of the body	1 (2.7)	0	0	1 (7.7)	0	0.594
THV-in-THV implantation	8 (21.6)	1 (5.9)	2 (66.7)	3 (23.1)	2 (50.0)	0.046
PPI	9 (24.3)	7 (41.2)	0	2 (15.4)	0	0.142
Annular rupture	0	0	0	0	0	-
Descending aortic dissection	2 (5.4)	0	0	2 (15.4)	0	0.272
Death	0	0	0	0	0	-
Moderate-to-severe PVL	5 (13.5)	0	0	4 (30.8)	1 (25.0)	0.076
Device success	25 (67.6)	16 (94.1)	1 (33.3)	6 (46.2)	2 (50.0)	0.016
Operation success	32 (86.5)	17 (100.0)	3 (100.0)	9 (69.2)	3 (75.0)	0.076

Note: THV = transcatheter heart valve; Oversize = 100 × (THV diameter/MDCT measurement diameter − 1); PPI = permanent pacemaker implantation; PVL = perivalvular leakage; * 21 patients before May 2021 are TAVR under general anesthesia and after May 2021, and 16 patients will receive TAVR under local anesthesia; ^#^ 28 patients before Aug 2021 are TAVR with first-generation non-recyclable THV systems and After Aug 2021, and 9 patients will receive TAVR second-generation recyclable THV systems because the second-generation recyclable THV systems were used in the China market on Aug 2021; a: comparison between types 2–4 and type 1; b: comparison between types 3–4 and type 2; c: comparison between types 4 and 3, *p* < 0.05.

**Table 5:** Changes in cardiac morphology and function during 1-year follow-up.

Characteristics (n,%)	Total N = 34	Type 1 N = 17	Type 2 N = 3	Type 3 N = 11	Type 4 N = 3	*p*
Left ventricular ejection fraction (%)						
Preoperative	53.1 ± 12.1	53.9 ± 10.1	41.3 ± 11.0	57.9 ± 12.3 ^b^	46.3 ± 16.3	0.120
Postoperative	48.9 ± 10.4	49.3 ± 11.0	44.0 ± 14.4	52.1 ± 8.2	42.5 ± 9.3	0.382
1 month	52.9 ± 9.5	54.7 ± 9.5	52.0 ± 13.5	51.4 ± 10.2	49.0 ± 7.2	0.701
3 months	52.4 ± 10.8	54.0 ± 10.2	47.0 ± 13.9	51.6 ± 13.0	52.7 ± 7.5	0.804
6 months	57.9 ± 8.8 ^n^	59.7 ± 5.2 ^n^	54.3 ± 15.7	57.0 ± 9.0	56.3 ± 15.3	0.798
1 year	59.0 ± 9.2 ^mnoq^	61.1 ± 7.5 ^mn^	54.3 ± 12.9	55.7 ± 10.1	61.7 ± 11.6 ^n^	0.511
Interventricular septal thickness (mm)						
Preoperative	10.2 ± 1.6	9.9 ± 1.5	8.7 ± 1.5	11.2 ± 1.2 ^ab^	10.0 ± 1.4	0.046
Postoperative	10.7 ± 1.7	10.7 ± 1.5	10.7 ± 2.9	11.6 ± 1.3	9.0 ± 1.8 ^c^	0.079
1 month	10.8 ± 2.4	10.4 ± 2.0	10.7 ± 3.1	12.0 ± 3.3	10.3 ± 1.0	0.466
3 months	10.7 ± 2.2	10.5 ± 2.1	10.0 ± 1.0	11.5 ± 3.0	9.7 ± 0.6	0.580
6 months	10.8 ± 1.8	10.5 ± 1.5	10.0 ± 2.0	11.5 ± 2.1	11.0 ± 2.6	0.655
1 year	10.3 ± 2.1	9.8 ± 1.4	8.7 ± 2.1	11.8 ± 3.0 ^b^	10.7 ± 1.5	0.133
Left ventricular end-diastolic diameter (mm)						
Preoperative	62.5 ± 7.3	61.4 ± 8.6	69.3 ± 4.0	62.7 ± 5.8	62.0 ± 4.7	0.393
Postoperative	56.4 ± 7.5 ^m^	54.7 ± 8.7 ^m^	60.3 ± 7.6 ^m^	56.4 ± 4.5 ^m^	61.3 ± 7.0	0.339
1 month	54.3 ± 6.9 ^m^	52.6 ± 6.5 ^m^	59.7 ± 6.0 ^m^	54.1 ± 4.9 ^m^	58.3 ± 11.0	0.251
3 months	52.9 ± 5.5 ^mn^	51.3 ± 4.1 ^m^	57.7 ± 3.2 ^m^	53.6 ± 6.7 ^m^	53.3 ± 8.5	0.338
6 months	50.1 ± 6.1 ^mno^	48.6 ± 6.8 ^mn^	56.7 ± 3.2 ^m^	49.5 ± 4.6 ^mn^	49.3 ± 6.7 ^m^	0.250
1 year	49.9 ± 6.4 ^mno^	48.8 ± 7.2 ^mn^	53.0 ± 5.2 ^m^	49.2 ± 6.0 ^mn^	53.0 ± 5.2	0.623
N-terminal pro–B-type natriuretic peptide (pg/mL)						
Preoperative	3481.4 ± 6419.5	2647.0 ± 5601.6	4594.7 ± 5298.8	1267.2 ± 1234.7	11,728.6 ± 12,265.7 ^ac^	0.034
Postoperative	1495.1 ± 1275.0 ^m^	1457.6 ± 1345.8	1932.3 ± 1580.9	1083.2 ± 863.0	2356.5 ± 1602.1 ^m^	0.369
1 month	1605.0 ± 1446.0 ^m^	755.1 ± 1145.0	2360.3 ± 3533.2	863.0 ± 817.0	1609.1 ± 1045.2 ^m^	0.294
3 months	934.4 ± 1293.8 ^m^	748.1 ± 1047.2	2050.0 ± 3026.0	710.2 ± 600.5	1070.5 ± 936.9	0.468
6 months	1038.3 ± 1862.9 ^m^	671.0 ± 762.7	2786.4 ± 4512.5	760.3 ± 1133.5	703.3 ± 679.0 ^m^	0.396
1 year	672.0 ± 929.3 ^m^	712.6 ± 1127.7	875.2 ± 1162.8	323.5 ± 342.6 ^m^	1383.5 ± 1130.7	0.539

Note: a: Types 2, 3, and 4 are compared with type 1; b: Types 3 and 4 are compared with type 2; c: Type 4 is compared with type 3; m: The follow-up period was compared with that before operation; n: The follow-up period was compared with that after operation; o: 3, 6, and 12 months after operation compared with 1 month after operation; q: 6 and 12 months after operation compared with 3 months after the operation, *p* < 0.05.

## Data Availability

The datasets used and/or analyzed during the current study are available from the corresponding author upon reasonable request.

## Supplementary Materials

The following supporting information can be downloaded at: https://www.mdpi.com/article/10.3390/jcm12031157/s1, Figure S1: TAVR-based CT dual-anchoring multiplanar measurement and anatomical classification of AR patients; Figure S2: Two self-expanding valve designs and dimensions.
