# Supplementary material for: Can Measuring the ‘Dual Anchors of Aorta’ Enhance the Success Rate of TAVR?—A Single-Center Experience
Source: J Clin Med. 2023 Feb 1;12(3):1157. doi: 10.3390/jcm12031157 (PMC9918180; doi:10.3390/jcm12031157)

Supplementary figures and legends

Figure S1. TAVR-based CT dual-anchoring multiplanar measurement and anatomical classification of AR patients.

AR=aortic regurgitation; CT=computed tomography; TAVR=transcatheter aortic valve replacement.

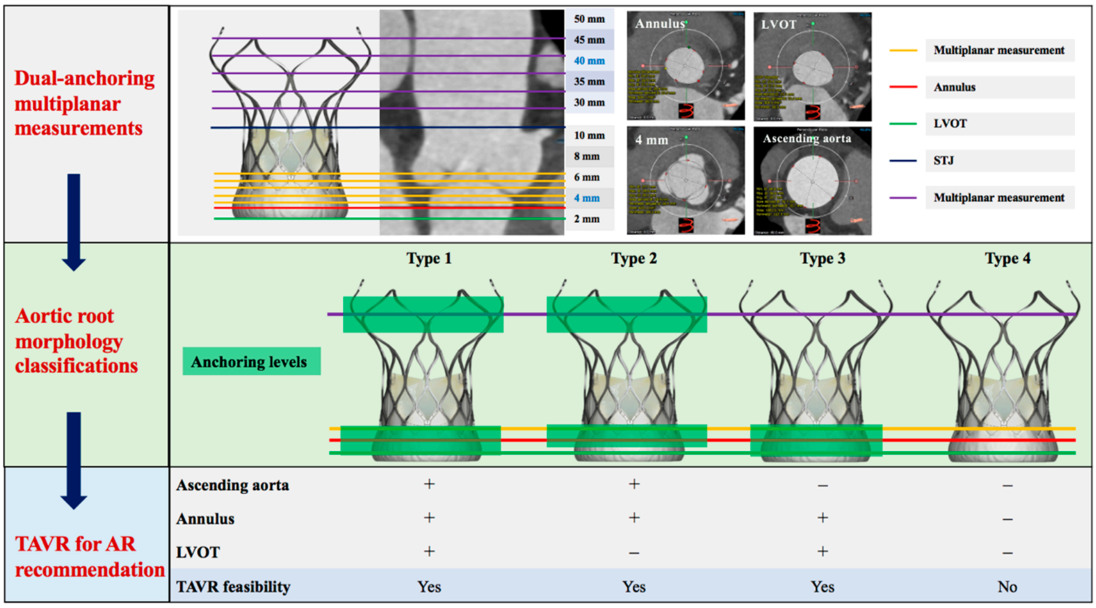

Figure S2. Two self-expanding valve designs and dimensions.

A: The Venus A valve is a self-expandable valve composed of a nitinol stent frame carrying a tri-leaflet bioprosthetic valve made of porcine pericardial leaflets. The Venus A prosthesis was available in 26 mm, 29 mm, and 32 mm sizes. B: The VitaFlow valve is made of a self-expanding nitinol frame and tri-leaflet bovine pericardial valve, which is characterized by low density and large cells at the ascending aorta part, high radial force, and outer polyethylene terephthalate skirt at the left ventricular outflow tract part. The VitaFlow prosthesis was available in 24mm, 27 mm, and 30 mm sizes.

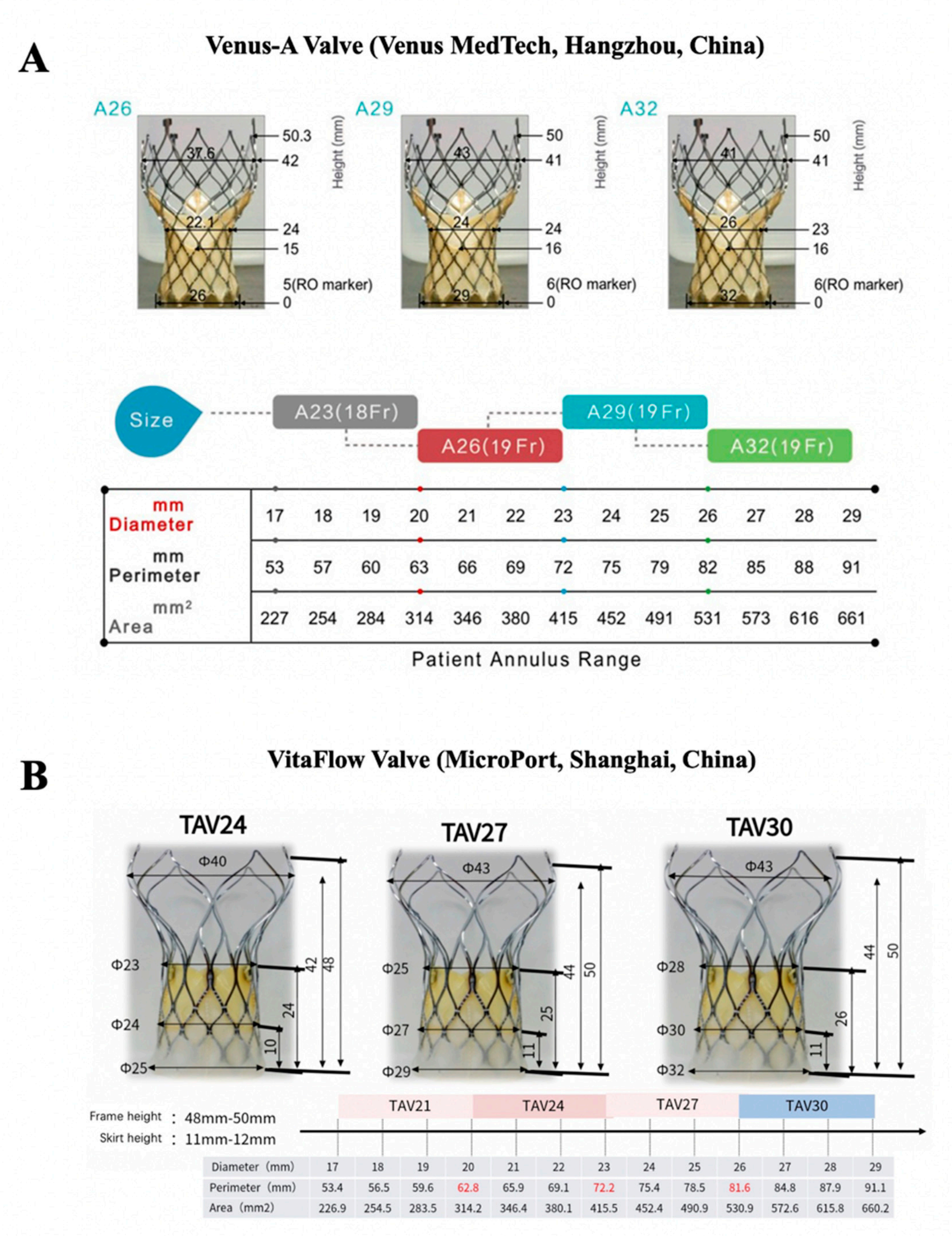

Supplement: Supplementary file 1 [file jcm-12-01157-s001.zip › jcm-2132925-supplementary.pdf]
